# Accurate information as a tool to decrease HIV test refusals in research studies

**DOI:** 10.2471/BLT.14.136515

**Published:** 2015-05-01

**Authors:** Susan C Watkins, Philip Anglewicz, Nicole Angotti, Amy Kaler, Ann Swidler

**Affiliations:** a California Center for Population Studies at UCLA, 4284 Public Affairs Building, MC 723603, PO Box 957236, Los Angeles, CA 90405, United States of America (USA).; b Tulane University School of Public Health and Tropical Medicine, New Orleans, USA.; c Department of Sociology and Center on Health, Risk and Society, American University, Washington, USA.; d Department of Sociology, University of Alberta, Edmonton, Canada.; e Department of Sociology, University of California, Berkeley, USA.

It has been argued that researchers conducting surveys that include testing for human immunodeficiency virus (HIV) have a duty to tell potential subjects that they do not have the right to participate if they refuse to receive their HIV test results.^1^^,^^2^ Furthermore, promotion of the routine feedback of such test results has been based on the grounds that knowledge is power and information is liberation.^3^ However, other researchers argue that, although it is desirable to offer study participants post-test counselling, for practical and ethical reasons some study participants should be given the right to refuse such counselling.^4^ Although we support the right of participants to opt out of post-test counselling and thus not to receive their test results, we also propose that subjects who are – or may be – tested for HIV should be given information that may decrease their resistance to learning their test results. We draw on data, collected between 1998 and 2013, on rural Malawians’ experience with – and perceptions of – HIV testing.

From the perspective of the organizations that promote HIV testing, it is axiomatic that people will benefit from knowing their HIV status. In Maher’s view, such benefit justifies sanctioning those who refuse to receive their test results.^2^ We disagree, for two reasons. First, the experience of many Malawians is that refusal to consent to testing may have serious consequences. For example, although the policy for antenatal HIV testing in Malawi includes an opt-out provision, accounts from pregnant women attending antenatal clinics show that HIV testing is compulsory if the women are to receive antenatal care.^5^ Moreover, in population-based HIV surveys, fieldworkers are always under pressure to minimize the numbers of test refusals and may exert undue pressure on individuals who do not want to receive their test results. While exclusion from antenatal services is, presumably, much more serious than exclusion from survey participation, in both of these examples people are sanctioned for not giving consent – which is a clear ethical violation.

Second, our ethnographic data depict the anguish that many suffer as they anticipate the future receipt of their test results – an issue that has rarely been discussed in the public health and social science literature.^6^ Two common misperceptions among rural Malawian adults are that the result of an HIV test will almost always be positive and that a positive result will inevitably be followed by hastening psychological deterioration, suicidal thoughts and death. Yet survey data from people living in rural Malawi show that between 80% and 90% of respondents who believed that they were HIV-positive before they were tested learned that they were HIV-negative.^7^^,^^8^

That so many are convinced, wrongly, that an HIV test will inevitably produce a positive diagnosis is the consequence of rural Malawians’ incorrect understanding of the probabilities of HIV transmission. For example, most of our survey respondents believed that an uninfected individual was certainly or highly likely to be infected with HIV during a single act of unprotected intercourse with an infected person.^8^ Would it not be preferable to treat those living amidst the HIV epidemic as having an ethical right to accurate information on the probabilities of transmission? Efforts should be made to evaluate the potential benefits of disseminating accurate information on the probabilities of transmission, the approximate prevalence of HIV infection and the probability of a positive result in an HIV test – such that consent for HIV testing in surveys is fully, rather than incompletely, informed. We need to know whether such health education would be a liberation, lead to fewer test refusals in research studies and, importantly, increase the number of people who are willing to know their HIV status.
